# The recombinase Rad51 plays a key role in events of genetic exchange in ***Trypanosoma cruzi***

**DOI:** 10.1038/s41598-018-31541-z

**Published:** 2018-09-06

**Authors:** Ceres Luciana Alves, Bruno Marçal Repolês, Marcelo Santos da Silva, Isabela Cecília Mendes, Paula Andrea Marin, Pedro Henrique Nascimento Aguiar, Selma da Silva Santos, Glória Regina Franco, Andréa Mara Macedo, Sérgio Danilo Junho Pena, Luciana de Oliveira Andrade, Alessandra Aparecida Guarneri, Erich Birelli Tahara, Maria Carolina Elias, Carlos Renato Machado

**Affiliations:** 1 Departamento de Bioquímica e Imunologia, Universidade Federal de Minas Gerais, Belo Horizonte, MG Brazil; 2 Laboratório Especial de Ciclo Celular, Centro de Toxinas, Resposta Imune e Sinalização Celular, Instituto Butantan, São Paulo, SP Brazil; 3 Departamento de Morfologia, Universidade Federal de Minas Gerais, Belo Horizonte, MG Brazil; 4 Centro de Pesquisas René Rachou, FIOCRUZ, Belo Horizonte, MG Brazil

## Abstract

Detection of genetic exchange has been a limiting factor to deepen the knowledge on the mechanisms by which *Trypanosoma cruzi* is able to generate progeny and genetic diversity. Here we show that incorporation of halogenated thymidine analogues, followed by immunostaining, is a reliable method not only to detect *T. cruzi* fused-cell hybrids, but also to quantify their percentage in populations of this parasite. Through this approach, we were able to detect and quantify fused-cell hybrids of *T. cruzi* clones CL Brener and Y. Given the increased detection of fused-cell hybrids in naturally-occurring hybrid CL Brener strain, which displays increased levels of RAD51 and BRCA2 transcripts, we further investigated the role of Rad51 – a recombinase involved in homologous recombination – in the process of genetic exchange. We also verified that the detection of fused-cell hybrids in *T. cruzi* overexpressing RAD51 is increased when compared to wild-type cells, suggesting a key role for Rad51 either in the formation or in the stabilization of fused-cell hybrids in this organism.

## Introduction

The parasitic protozoan *Trypanosoma cruzi* is the causative agent of Chagas disease, which is present in more than twenty countries in the Americas, currently affecting eight to ten million people^1^. Similarly to other members of the Trypanosomatidae family, *T. cruzi* is a pathogen that exhibits a complex life cycle, involving both vertebrate and invertebrate hosts^2,3^.

Since the discovery of Chagas disease, there have been heated debates on *T. cruzi* reproductive mode as it is not well established yet if its progeny is generated by preponderate clonal evolution, or if cryptic events of genetic exchange promoted by sexual reproduction – which would lead to the occurrence of natural hybrids – could possibly play a role in this process. In fact, despite of various studies on the topic, many fundamental aspects about the mechanisms by which *T. cruzi* cells reproduce remain unknown to date^4,5^.

It is well accepted that the wide array of clinical manifestations of Chagas disease is related to hosts genetics and environment factors, as well as to the great genetic variability observed among *T. cruzi* genetic groups^6^. Currently, these genetic groups are divided into six discrete typing units (DTUs), or clades, namely TcI to TcVI. The *T. cruzi* classification in DTUs is based on molecular markers, geographic distribution, epidemiological associations, and clinical manifestations^7–10^. Interestingly, with the attempt to clarify the evolutionary relations between all these groups, evidence was found suggesting the natural occurrence of genetic exchange in some of them. TcV and TcVI were identified as hybrids originated from TcII and TcIII^4,11–14^. Moreover, back in 2003, Gaunt *et al*. were able to identify events of genetic exchange by detecting the presence of *T. cruzi* fused-cell hybrids isolated from the mammalian host carrying two different drug-resistance markers (neomycin and hygromycin B), each one coming from distinct populations of *T. cruzi* I^12^, suggesting that genetic exchange could take place in specific life cycle phases^12^. However, it is not clear yet if the mechanisms of such genetic exchange in *T. cruzi* are similar to those observed in other parasites such as *Leishmania* and *T. brucei*, which present sexual recombination driven by meiosis^15,16^.

Mechanisms of reproduction and the extent of clonality of a variety of organisms have been addressed^17^. Currently, although mechanisms of genetic exchange during life cycle are believed to be essential to provide the progeny genetic variability which confers adaptability to the environment^18^, there are no consensus to which extent this phenomenon may influence the population genetics of a given species. Based on studies relying on linkage disequilibrium and Hardy-Weinberg deviations, a model of clonal reproduction with rare events of genetic recombination is accepted to explain genetic exchange in this trypanosomatid^9,18–23^. Also, recent investigations about genetic exchange in *T. cruzi* have provided insights about its impact onto populations of this parasite: analysis using allele frequency and haplotype networks in different loci of isolated *T. cruzi* populations from the Brazilian state of Minas Gerais demonstrated that genetic exchange may be more frequent than originally expected as a linkage disequilibrium was found in Latin-American populations of *T. cruzi* opposed to other small geographic areas^9^; also, investigation using 49 microssatellites loci demonstrated the existence of an excess of heterozygosity for some *T. cruzi* DTU’s^24,25^. The same was observed in a study using isolates from Ecuador, which described linkage disequilibrium in physically linked loci^26^. In fact, there is sufficient evidence to support the occurrence of genetic recombination in laboratory and free-living *T. cruzi* strains, as recently revised by Messenger and Miles^4^.

However, trypanosomatids predominantly reproduce by clonal generation through longitudinal binary fission, and not through sexual reproduction; in this sense, mutations and parasexual exchange are currently the most parsimonious models to explain genetic variability in *T. cruzi*^4^. Interestingly, parasexual exchange has already been described in some fungi^27^, and is characterized by DNA exchange that allows genetic recombination without either meiosis, or gamete formation, or even the necessity of fertilization. Since there is no demonstration of gamete formation in *T*. *cruzi* so far, parasexual reproduction could be important for the heterogeneity observed amongst different populations of this parasite – indeed, a recent analysis of 45 sequenced *T. cruzi* genomes from TcI DTU shows that both clonal expansion and parasexual reproduction are important to provide genetic variability^28^.

A major mechanism that could be co-related to the parasexual exchange is homologous recombination (HR), which can occur throughout the genome^29^. In fact, HR is able to produce new combinations of nucleotide sequences, generating genetic diversity and, in some cases, cell hybrids^30–32^. In addition, HR is the most important pathway for *T. cruzi* to cope with DNA double-strand breaks (DSBs) as essential proteins for the non-homologous end joining pathway have not been identified in the genome of this organism^32^. Interestingly, *T. cruzi* presents a high resistance against ionizing radiation, a genotoxic agent which is an important source of DSBs^33,34^ this peculiar phenotype most certainly a consequence of an efficient HR DNA repair pathway^34–36^. One important protein involved in this pathway is the recombinase Rad51, whose role is to search for homology during the DNA repair process^37^. In fact, in mammalian cells, once the genomic lesion is recognized, (i) DNA ends are processed by the Mre11-Rad50-Nbs1 (MRN) complex, (ii) oligomers of Rad51 are disrupted by BRC domains found in tumor suppressor gene BRCA2, and then (iii) Rad51 is loaded onto single-stranded DNA, being the formation of this nucleoprotein filament one of the essential steps to repair DSBs^37,38^. Also, it has already been demonstrated that the overexpression of Rad51 in *T. cruzi* can alter the dynamic of DNA repair conducted by HR: after being exposed to the dose of 500 Gy of ionizing gamma radiation, *T. cruzi* cells overexpressing RAD51 are able to resume their cellular growth earlier than wild-type cells; this phenotype is probably related to the enhanced capability that RAD51-overexpressing parasites exhibit to repair DSBs generated by this genotoxic agent^35^.

In this study, we show that naturally-occuring hybrid *T. cruzi* clone CL Brener presented decreased cellular arrest in response to ionizing irradiation, higher basal levels of RAD51 and BRCA2 transcripts, and increased detection of cellular genetic exchange when compared to naturally-occurring non-hybrids. In addition, we show that overexpression of RAD51 in *T. cruzi* further increased the detection of fused-cell hybrids, which present higher ploidy, impaired growth rate, but decreased cellular arrest due to ionizing radiation. Collectively, our data suggest that the HR pathway, through the activity of the recombinase Rad51, is involved either in the formation or in the stabilization of *T. cruzi* fused-cell hybrids, playing an important role to the generation of intraspecific variability and environmental adaptation of this trypanosomatid.

## Results

### Naturally-occurring T. cruzi hybrids present improved response against ionizing radiation, and increased levels of RAD51 and BRCA2 transcripts

**Figure 1:**
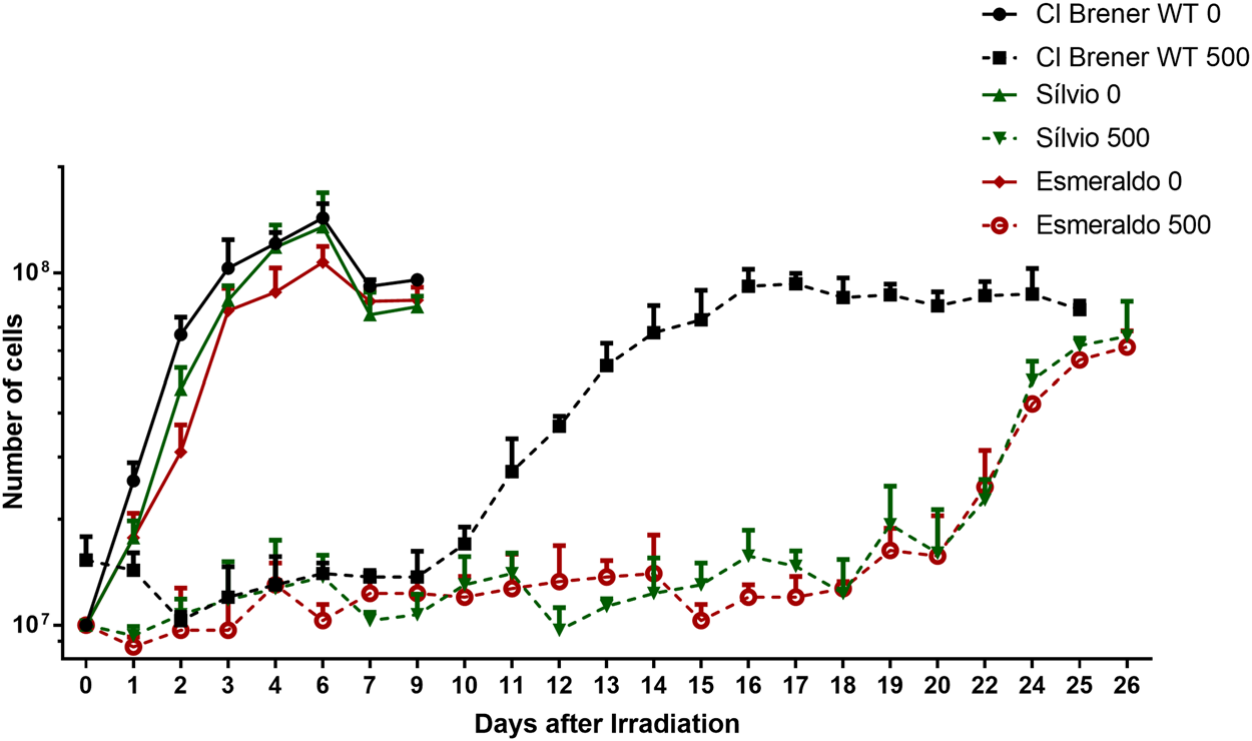
Naturally-occurring *T. cruzi* hybrids present improved response against ionizing radiation. CL Brener, Esmeraldo and Sylvio epimastigotes were exposed to 500 Gy of ionizing radiation, which generate DNA DSBs. The naturally-occurring hybrid CL Brener strain showed decreased cellular growth arrest when compared to naturally-occurring non-hybrid strains Esmeraldo and Sylvio.

One of the biological effects promoted by ionizing radiation is the generation of DNA DSBs^33^. It is known that epimastigotes of *T. cruzi* clone CL Brener – naturally-occurring hybrids – survive to doses of ionizing radiation as high as 1500 Gy, and, after being exposed to the dose of 500 Gy, remain in lag phase for 10 days – at the end of which they resume their replicative growth, even though the majority of damaged DNA gets repaired after 48 h^35^. In order to verify whether the elapsed time taken to resume *T. cruzi* logarithmic growth upon exposure to ionizing radiation differed between naturally-occurring non-hybrid strains and CL Brener – this may indicate the existence of intra-specific variations with regard to the efficiency of DNA repair processes in this parasite –, we treated epimastigotes from naturally-occurring non-hybrid strains – namely Sylvio (TcI) and Esmeraldo (TcII) – in parallel with epimastigotes from CL Brener strain with the dose of 500 Gy of ionizing radiation. As shown in Fig. 1, all three strains presented a stationary phase following irradiation; however, unlike CL Brener epimastigotes, which resumed their cellular growth after 10 days as previously described^34–36^, Sylvio and Esmeraldo epimastigotes exhibited longer stationary phases as their cellular growth resumed only 15 days after exposure to ionizing radiation, showing that CL Brener cells present decreased cellular growth arrest in response to genotoxicity. The same growth recovery pattern was observed when other naturally-occurring hybrid strain Bug2149 cl10 (TcV), and non-hybrid strains Dm28c (TcI) and Y (TcII) were analyzed (S1 Fig.).

**Figure 2:**
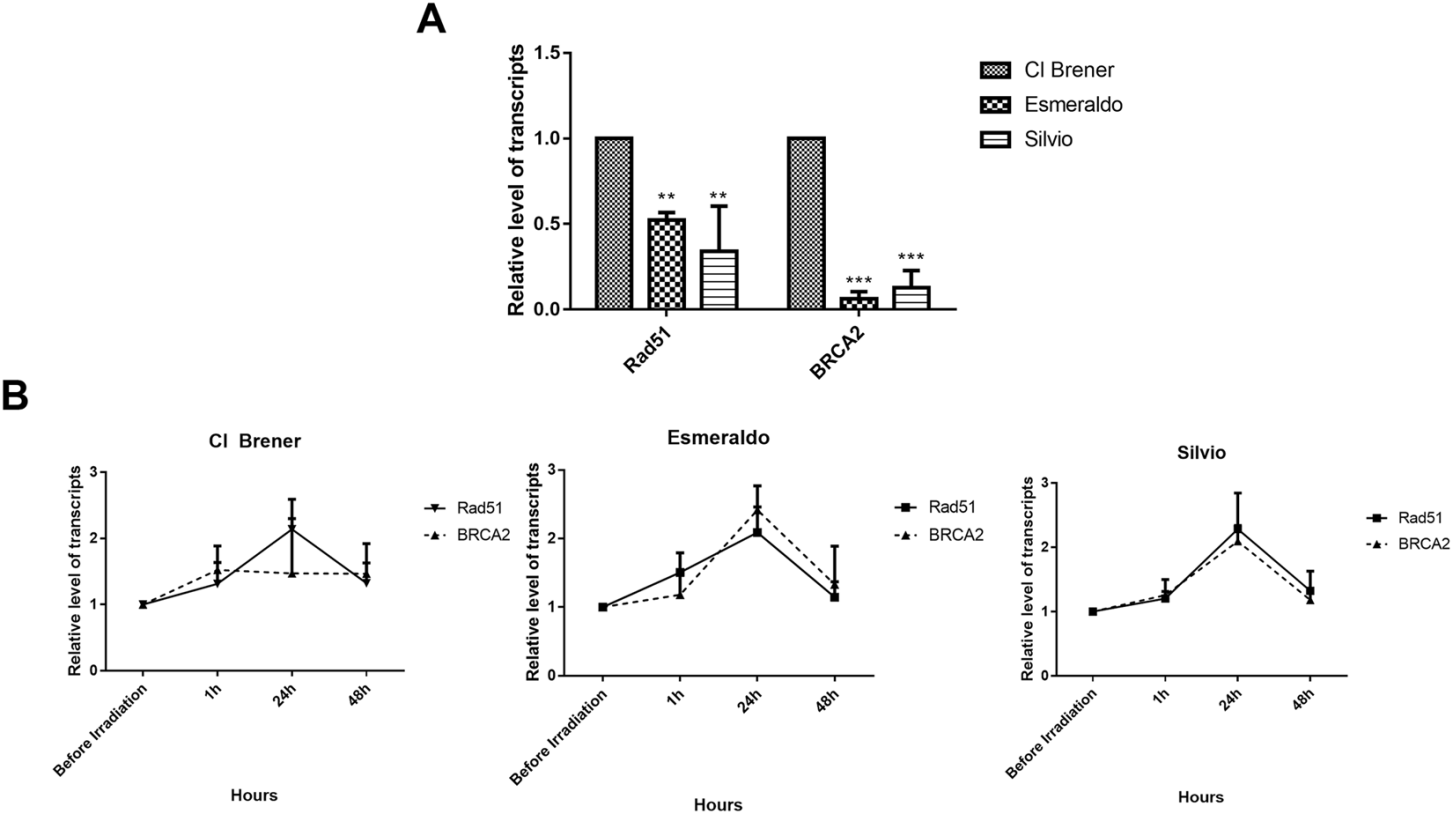
Naturally-occurring *T. cruzi* hybrids present increased levels of RAD51 and BRCA2 transcripts. (**A**) Naturally-occurring non-hybrid Esmeraldo and Sylvio strains exhibit lower basal levels of RAD51 and BRCA2 transcripts when compared to Cl Brener strain. (**B**) After irradiation, levels of RAD51 and BRCA2 transcripts increased in all strains. Transcription of RAD51 and BRCA2 present highest levels at 24 h. The comparison was made with the respective non-irradiated strain at each time analyzed. Experiments were performed in triplicate. *p < 0.05, **p < 0.01 and ***p < 0.001 vs. _ (One-way ANOVA).

The fact that hybrid strains epimastigotes, after exposure to ionizing radiation, resumed their cellular growth earlier than all naturally-occurring non-hybrid strains tested (Fig. 1; S1 Fig.) suggests that naturally-occurring hybrids present a more efficient repair mechanism of DSBs. We then hypothesized that genes involved in DSB repair pathway could be differentially transcribed in all *T. cruzi* strains previously examined. In order to test this hypothesis, we determined the levels of RAD51 and BRCA2 transcripts in epimastigotes through real-time quantitative PCR, using specific primers to each gene. As shown in Fig. 2A, transcriptional levels of RAD51 and BRCA2 are higher in the CL Brener strain; in fact, the shorter cellular arrest exhibited by this naturally-hybrid strain may be associated to the increased basal levels of both Rad51 and Brca2 as Esmeraldo and Sylvio strains – which display lower transcriptional levels of RAD51 and BRCA2 when compared to CL Bener –also exhibited the same patterns of induction of transcription for these two genes after ionizing radiation treatment (Fig. 2B). Nonetheless, Sylvio and Esmeraldo epimastigotes showed lower absolute levels of both RAD51 and BRCA2 transcripts when compared to CL Brener cells, in all time points analyzed, regardless if ionizing radiation treatment was conducted or not (Fig. 2).

### Increased co-detection of halogenated thymidine analogues in naturally-occurring *T. cruzi* hybrids showed increased rates of genetic exchange and formation of fused-cell hybrids

**Figure 3:**
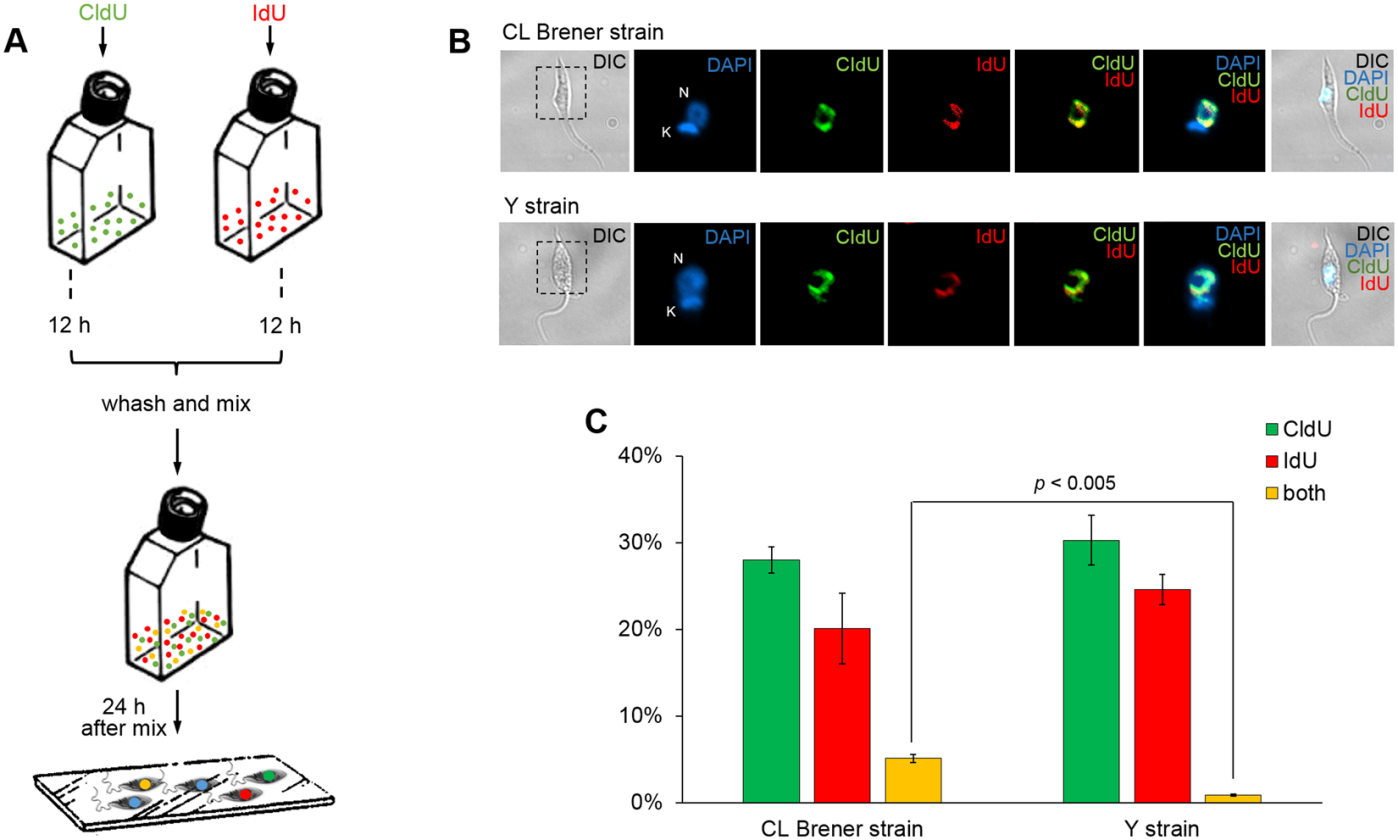
Increased co-detection of thymidine analogues in naturally-occurring *T. cruzi* hybrids shows increased rates of genetic exchange and formation of fused-cell hybrids. (**A**) Schematic diagram representing thymidine analogues incorporation assay. Thymidine analogues (CldU and IdU) were separately incubated in each culture for 12 h. After that, cells of *T. cruzi* clones CL Brener and Y were washed and pooled together, remaining mixed for 24 h. Samples were then added onto slides, and the process for analogues detection (using α-CldU/α-IdU, and corresponding secondary antibodies) was performed. (**B**) Representative images were organized in seven columns: DIC (differential interference contrast), depicting morphology; DAPI, depicting staining nucleus (N) and kinetoplast (K); CldU, depicting CldU incorporation; IdU, depicting IdU incorporation; and CldU/IdU, DAPI/CldU/IdU, and DIC/DAPI/CldU/IdU depicting CldU/IdU, DAPI/CldU/IdU and DIC + DAPI + CldU + IdU overlays, respectively. All images represent fused-cell hybrids from clones CL Brener and Y. Images were analyzed using Olimpus BX51 fluorescence microscope, and captured randomly to avoid bias. (**C**) Percentage of *T. cruzi* cells labeled with CldU (green), IdU (red), or with both analogues (yellow). The total of 233.6 ± 16.7 and 267 ± 6 cells of CL Brener and Y, respectively, were analyzed. Experiments were carried out in triplicate, and error bars indicate standard deviation. Statistical analysis was performed using Student’s *t*-test.

In order to experimentally check, under laboratory conditions, the possibility of assessing the rates of genetic exchange in *T. cruzi* cells, we developed an experimental assay based on cellular incorporation of two distinguishable halogenated thymidine analogues, namely 5′-chloro-2′-deoxyuridine (CldU) and 5′-iodo-2′-deoxyuridine (IdU), whose presence in the genetic material can be detected by immunostaining through the use of specific antibodies that generate distinct signals – red for CldU, and green for IdU – in labeled cells. As described in *Materials and methods*, and represented in Fig. 1A, either CldU or IdU was separately added to cultures of epimastigotes from two *T. cruzi* strains elected for this assay – naturally-occurring hybrid CL Brener strain (TcVI), and naturally-occurring non-hybrid Y strain (TcII)^8^. After 12 h in the presence of either CldU or IdU, *T. cruzi* cells were then washed thoroughly to eliminate the residual content of unincorporated halogenated thymidine analogues. Then, both CldU- and IdU-labeled cells from the same strain were co-incubated in the same culture flask, remaining together for the following 24 h (Fig. 3A). Based on the fact that CldU- and IdU-labeled cultures would not be able to incorporate the analogue they were not previously incubated with, simultaneous detection of both halogenated thymidine analogues in a given cell would be a result of genetic exchange and formation of fused-cell hybrids. Interestingly, after 24 h of co-incubation of CldU- and IdU-labeled cells, cultures from both *T. cruzi* clones – CL Brener and Y – exhibited epimastigotes harboring nuclear co-localization of the two thymidine analogues (Fig. 3B), showing the suitability of this experimental approach to determine genetic exchange in this parasite. Possible cross-reactions between the antibodies used in this assay – α-CldU and α-IdU primary antibodies, and Alexa Fluor 488 and Alexa Fluor 555 secondary antibodies – were ruled out as immunostaining using α-CldU against IdU-labeled parasites, and α-IdU against CldU-labeled ones, showed no presence of any detectable signals (S2 Fig.). The observed average percentage of epimastigotes co-localizing CldU and IdU in relation to the total cellular population, from three independent experiments, was 5.1% in CL Brener strain, and 0.85% in Y strain (Fig. 3C). The difference between those averages was statistically significant (*p* < 0.005, Student’s *t* test), confirming the hypothesis that genetic exchange rates in *T. cruzi* clone CL Brener, a naturally-occurring hybrid strain, are significantly higher than those observed in *T. cruzi* clone Y, a naturally-occurring non-hybrid strain. Also, to rule out the possibility that the double labeled cells were an artifact due to insufficient washing prior mixing, cells were washed extensively on high volumes of PBS. No significative alterations were observed on the percentage of double-stained cells (Fig. S2).

### Overexpression of RAD51 increases the detection of *T. cruzi* fused-cell hybrids

**Figure 4:**
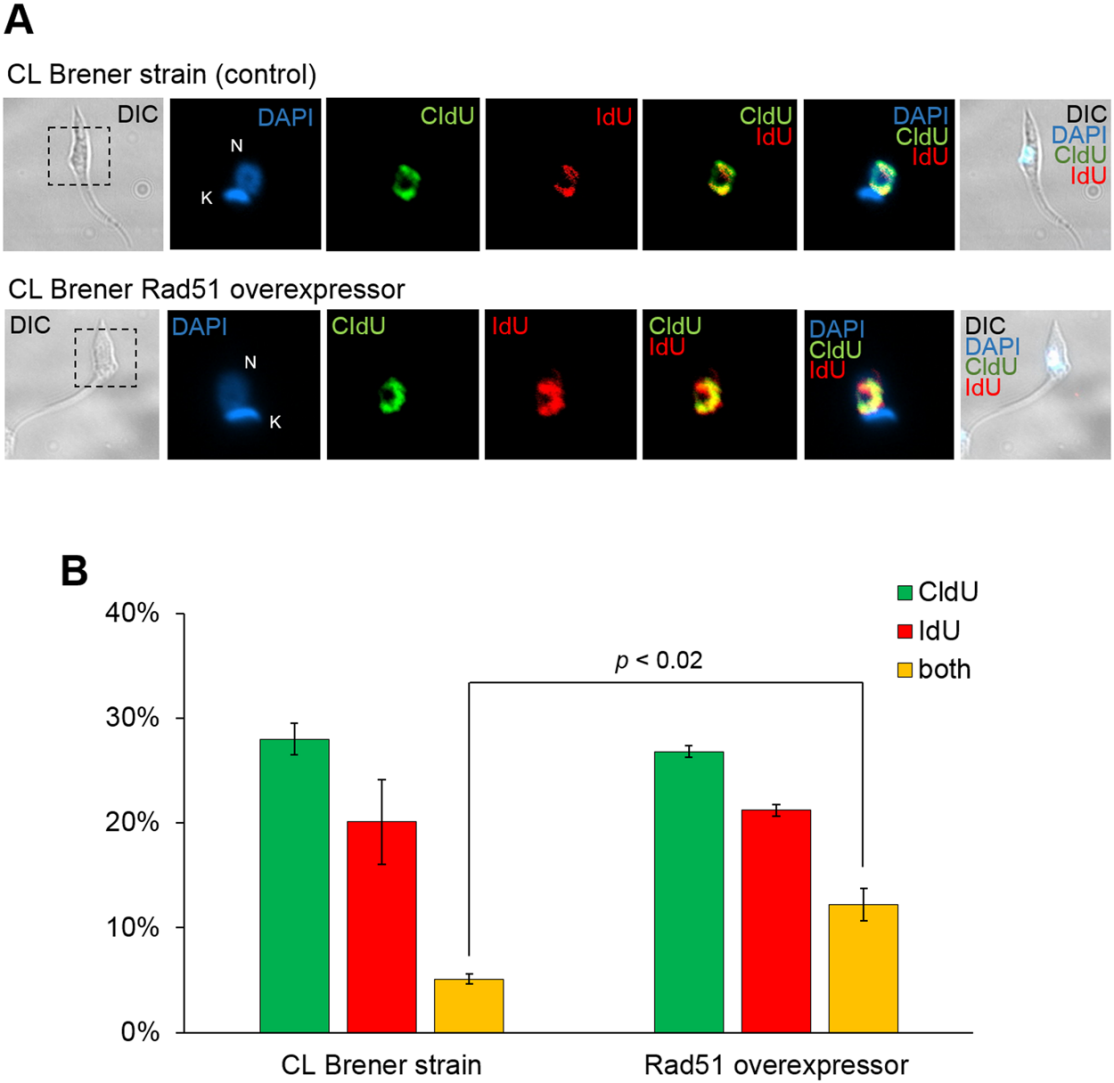
Overexpression of RAD51 increases the detection of *T. cruzi* fused-cell hybrids. (**A**) Representative images were organized in seven columns: DIC (differential interference contrast), depicting morphology; DAPI, depicting staining nucleus (N) and kinetoplast (K); CldU, depicting CldU incorporation; IdU, depicting IdU incorporation; and CldU/IdU, DAPI/CldU/IdU, and DIC/DAPI/CldU/IdU depicting CldU/IdU, DAPI/CldU/IdU and DIC + DAPI + CldU + IdU overlays, respectively. All images represent fused-cell hybrids from clones CL Brener and Y. Images were analyzed using Olimpus BX51 fluorescence microscope, and captured randomly to avoid bias. (**B**) Percentage of *T. cruzi* cells labeled with CldU (green), IdU (red), or with both analogues (yellow). The total of 233.6 ± 16.7 and 231 ± 23.5 cells of CL Brener and Y, respectively, were analyzed. Experiments were carried out in triplicate, and error bars indicate standard deviation. Statistical analysis was performed using Student’s *t*-test.

Aiming to investigate if the increased rates of genetic exchange observed in CL Brener strain (Fig. 3) were related to increased levels of RAD51 and BRCA2 transcription levels (Fig. 2), we sought to investigate whether components from the recombination machinery could exert a direct effect on the detection of *T. cruzi* fused-cell hybrids. For such, we decided to construct a *T. cruzi* clone of CL Brener overexpressing RAD51, a key component of the recombination machinery^37^. Making use of the thymidine incorporation assay previously described (Fig. 3), we determined the percentage of wild-type and RAD51-overexpressing *T. cruzi* clone CL Brener epimastigotes showing nuclear co-localization of both thymidine analogues (Fig. 4). After three independent assays, we found that the percentage of fused-cell hybrids generated by overexpression of RAD51 was 12.2%, compared to 5.1% in wild-type epimastigotes (Fig. 4B). The difference between those averages was statistically significant (*p* < 0.02, Student’s *t* test), and shows that genetic exchange rates in *T. cruzi* epimastigotes overexpressing RAD51 are higher than in control cells; in fact, RAD51-overexpressing cells exhibit a more evident co-localization of both analogues (Fig. 4A). Taken together, these results suggest an important involvement of Rad51 in genetic exchange process in *T. cruzi*, either in the formation of fused-cell hybrids, or in the stabilization of them.

### Axenic culture is capable of generating *T. cruzi* fused-cell hybrids

**Figure 5:**
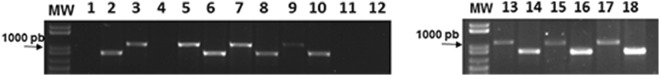
Axenic culture is capable of generating *T. cruzi* fused-cell hybrids. Fused-cell hybrids isolated from axenic cultures exhibited hygromycin B and neomycin genes amplified after genomic DNA extraction and PCR. Parental cells showed only one gene being amplified. The figure represents two separated runs in different gels. MW: 1 Kb Plus DNA Ladder; 1: pRock-neo DNA + hygromycin B primers; 2: pRock-neo DNA + primer neomycin primers; 3: pRock + RAD51-hygromycin B DNA + hygromycin primers; 4: pRock + RAD51-hygromycin B + neomycin primers; 5: clone #1 DNA + hygromycin B primers; 6: clone #1 DNA + neomycin primers; 7: clone #2 DNA + hygromycin B primers; 8: clone #2 DNA + neomycin primers; 9: clone #3 DNA + hygromycin B primers; 10: clone #3 DNA + neomycin primers; 11: negative control (DNA absent) + hygromycin B primers; 12: negative control (DNA absent) + neomycin primers; 13: clone #4 DNA + hygromycin B primers; 14: clone #4 DNA + neomycin primers; 15: population #1 DNA + hygromycin B primers; 16: population #1 DNA + neomycin primers; 17: population #2 DNA + hygromycin B primers; 18: population #2 + neomycin primers.

**Figure 6:**
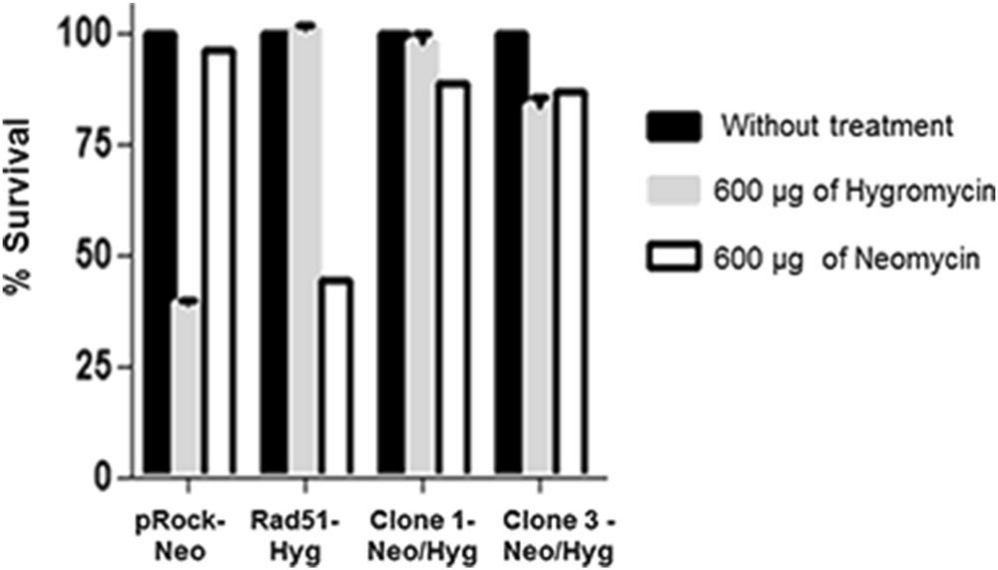
*T. cruzi* fused-cell hybrids present increased survival rate after co-treatment with hygromycin B or neomycin. Fused-cell hybrids survival rates were assessed 72 h after treatment with hygromicin B and/or neomycin. Fused-cell hybrids are resistant to both hygromycin B and neomycin, and parental cells (harboring only hygromycin B or neomycin resistant genes) showed marked sensitivity when co-incubated with both antibiotics.

In order to further characterize the generation of fused-cell hybrids and the importance of Rad51 towards genetic exchange in *T. cruzi*, we decided to verify whether fused-cell hybrids could be recovered from (i) an axenic culture, (ii) a mammalian cell culture, and (iii) triatomine bugs, as described in *Materials and methods*. While no resistant parasites against hygromycin B and neomycin were recovered from the passages conducted either in mammalian cell cultures or in triatomine bugs, the solely axenic *T. cruzi* epimastigote cultures allowed us to recover two populations of fused-cell hybrids simultaneously carrying resistance genes to both antibiotics (Fig. 5). Two different clones were obtained from each of the two populations of fused-cell hybrids, and, through PCR, we verified that the isolated clones (clones #1 to #4 of te first gel) – resistant to both antibiotics – indeed co-exhibited the hygromycin B and neomycin genes (Fig. 5). It is important to stress that no fused-cell hybrids were obtained from experiments performed with a pool of *T. cruzi* cells with either pROCK NEO or pROCK HYG. We also decided to assess the survival rate of two clones (#1 and #3) 72 h after treatment with 600 μg/mL hygromicin B and/or 600 μg/mL neomycin, and verified that both clones showed resistance against concentrations three-fold higher than usual for both antibiotics, while the two parental cells – those harboring either only RAD51 and hygromycin-resistant genes, or only the neomycin-resistant one – were sensitive to treatment with the antibiotics (Fig. 6).

### Fused-cell hybrids present increased ploidy, impaired growth, but improved response against ionizing radiation

**Figure 7:**
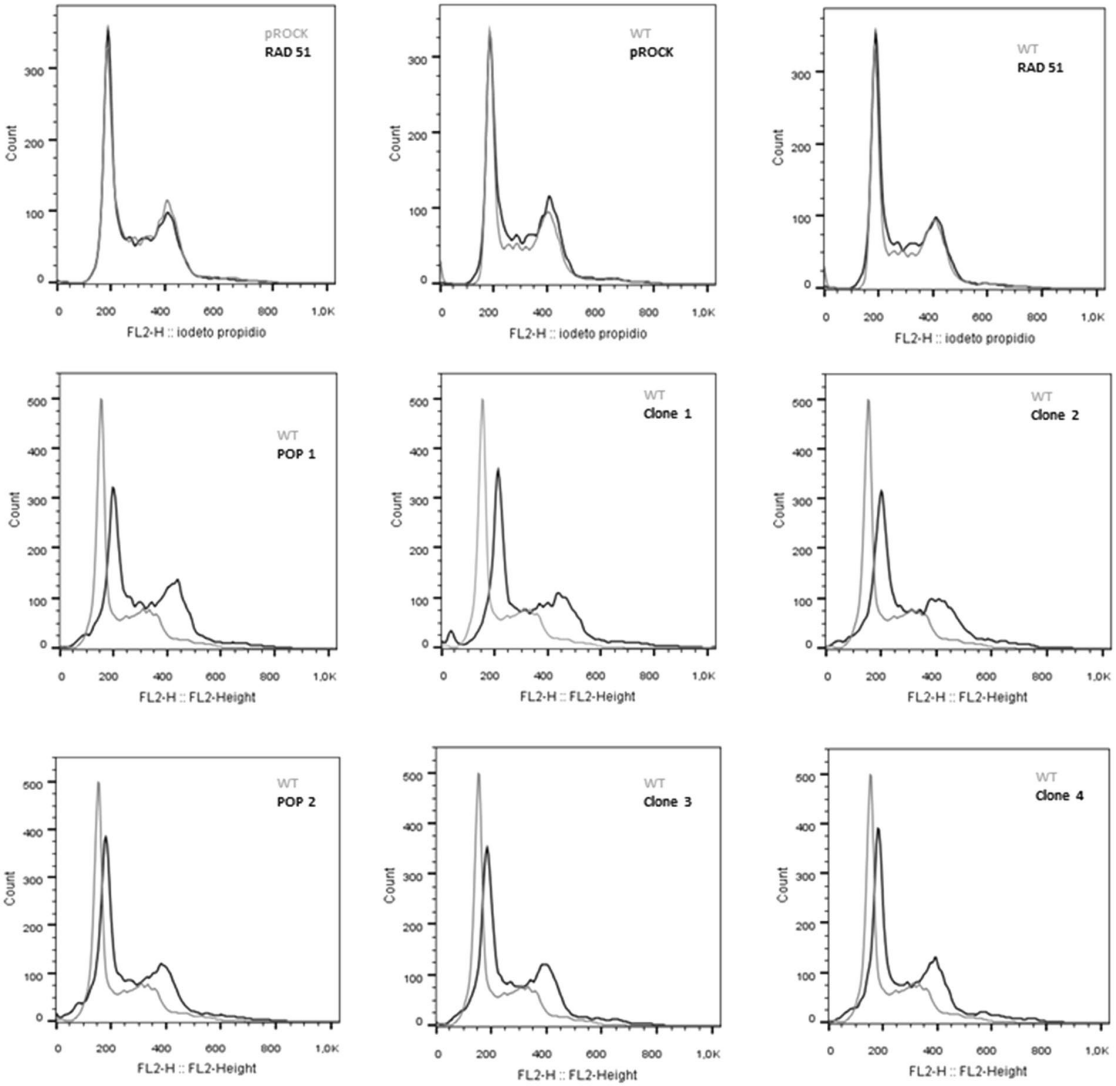
Fused cell hybrids present increased ploidy. Overlaid histograms show that the content of DNA from *T. cruzi* fused-hybrid cells is increased. pRock + RAD51: parental cells; POP: population; WT: wild-type; pRock: cells carrying pRock empty vector; RAD51: RAD51-overexpressing cell.

**Figure 8:**
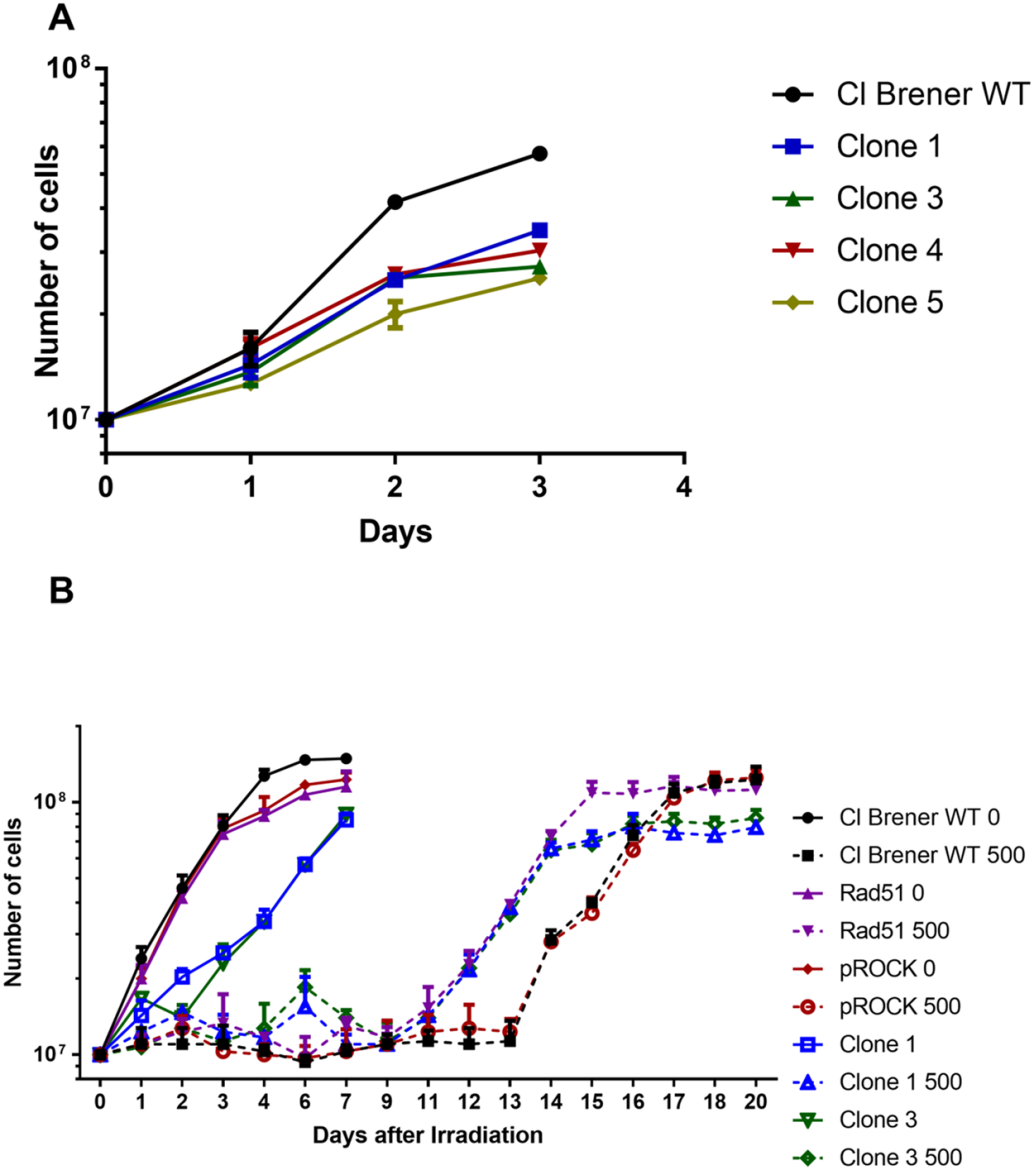
Fused cell hybrids present impaired growth, but decreased cellular growth arrest in response to ionizing radiation. (**A**) CL Brener Fused-cell hybrids (clones #1 to #5) show decreased growth when compared to non-fused cells (WT). (**B**) Fused-cell hybrids (clones #1 and #3) present decreased cellular growth arrest after being exposed to 500 Gy of ionizing radiation.

Fused-cell hybrids from *T. cruzi* were further characterized through the determination of their ploidy by flow cytometry, using the fluorescent DNA binding dye propidium iodide. While flow cytometry analysis showed that total DNA content does not vary between untransfected epimastigotes and epimastigotes overexpressing RAD51, the ploidy of fused-cell hybrids is evidently increased (Fig. 7). In addition, fused-cell hybrids (clones #1 to #5) showed decreased growth rates in comparison to the unstransfected CL Brener epimastigote (Fig. 8).

As previously presented, after being exposed to moderate doses of ionizing radiation, *T. cruzi* epimastigotes exhibit a lag phase, and resume their cellular growth several days after irradiation, being this lag phase shorter when parasites are naturally-occurring hybrids (Fig. 2A)^34–36^. We then finally investigated if fused-cell hybrids would be able to resume their cellular growth, after exposure to ionizing radiation, earlier than non-fused cells. Interestingly, after being treated with the dose of 500 Gy, fused-cell hybrids present decreased cellular growth arrest. These cells were able to resume cellular growth earlier than non-fused cells, and as fast as RAD51-overexpressing cells (Fig. 8).

## Discussion

Reproductive mechanisms of the trypanosomatid *T. cruzi* have been subject of intense discordance^4,5^. Although it is accepted that genetic recombination possibly played a role at an evolutionary scale, the idea that this parasite undergoes hybridization to a relevant extent, at a generational scale, is still under some disbelief^39,40^. Interestingly, the fact that *T. cruzi* clone CL Brener presents a proficient mechanism of DNA DSBs repair suggests that this strain is undoubtedly prone to efficiently perform HR^34–36,41,42^. Since HR has been described as an important factor to generate genetic exchange and promote environmental fitness in all domains of life^43–48^, we then hypothesized that *T. cruzi* CL Brener harbors a strong prerogative which qualifies it as a potential subject of genetic exchange.

In fact, when compared to naturally-occurring non-hybrid strains, naturally-occurring hybrid epimastigotes present decreased cellular growth arrest after exposure to gamma radiation (Figs 1 and S1). CL Brener strain presents higher levels of RAD51 and BRCA2 transcripts (Fig. 2). Brca2 is a protein involved in Rad51 loading into the broken DNA site in order to promote the formation of the nucleoproteic filament required to HR^37,49,50^. Rad51 is responsible for strand invasion and search of homology between the broken strand and the template^37,51^. Also, it has already been demonstrated that particular variations in HR can arise from alterations in Rad51 and Brca2 activities^52,53^. RAD51-silenced *Trypanosoma brucei* exhibits defective variant surface glycoprotein (VSG) switching and increased tendency to the generation of DSBs^54^; these phenotypes are also observed in *T. brucei* strains carrying mutations that reduce the number of BRC repeats in BRCA2^55^. Although RNA abundance is not generally indicative of protein levels in *T. cruzi*, the faster recovery shown by CL Brener – a naturally-occurring hybrid strain – to resume its cellular growth when compared to naturally-occurring non-hybrid strains (Fig. 1; S1 Fig.) may be related with the difference of RAD51 and BRCA2 transcriptional levels (Fig. 2) since the interaction of Rad51 with Brca2 is essential to an efficient cellular response to DSBs generation^41,56^.

We next developed an assay based on thymidine analogues incorporation, aiming to detect possible *T. cruzi* fused-cell hybrids through immunostaining. Interestingly, we were not only able to detect cells that underwent genetic exchange in *T. cruzi* clones CL Brener and Y, but also to quantitate the percentage of the sub-population that has been subject of this process (Fig. 3). Remarkably, the naturally-occurring hybrid CL Brener strain shows a significant increase in the percentage of epimastigotes that went through genetic exchange (5,1% ± 0,47) when compared to the naturally-occurring non-hybrid strain Y (0,85% ± 0,13). This result suggested that (i) genetic exchange in *T. cruzi* may be more pervasive than initially believed, and that (ii) there is a correlation between the rates of genetic exchange detection and the expression levels of RAD51 and BRCA2. We then sought to use a *T. cruzi* clone CL Brener overexpressing RAD51 in an attempt to verify whether a further increase in Rad51 levels could further increase the frequency of detection of genetic exchange in this parasite. Surprisingly, the detection of fused-cell hybrids in CL Brener epimastigotes overexpressing RAD51 exhibited significant increase in relation to wild-type CL Brener epimastigotes (Fig. 4), showing that Rad51 plays an important role in events of genetic exchange in *T. cruzi*, either in the generation or in the stabilization of fused-cell hybrids.

In order to further characterize the process of formation and isolation of fused-cell hybrids, we next determined whether fused-cell hybrids could be obtained from axenic cultures, mammalian cells or triatomine bugs. Contrarily to Gaunt *et al*., who obtained fused-cell hybrids after a passage in mammalian cells^12^, we were able to recover fused-cell hybrids solely from axenic cultures (Figs 5 and 6). We hypothesize that in the work from Gaunt *et al*. genetic exchange may have been allowed to occur during the conversion of epimastigotes into trypomastigotes in axenic cultures, being the passage in mammalian cells a selective pressure for isolation of fused-cell hybrids. Also, noteworthy is the fact that other trypanosomatids, namely *Leishmania spp*. – which present natural hybrids^57,58^ –, and *T. brucei* – which is able to form haploid gametes^16^ –, exhibit a sexual cycle in the insect vector, suggesting that, in nature, this is the environment in which genetic exchange takes place^15,16,59–62^. We further hypothesize that our group was not able to detect fused-cell hybrids in the triatomine bug as the total period of passage in the insect vector – 30 d – was not long enough to allow genetic exchange, or stabilization of fused-cell hybrids. Finally, as expected, fused-cell hybrids from axenic cultures show predicted inheritance of traits from parental cells once they present co-resistance against hygromycin B and neomycin (Fig. 6).

Ploidy of fused-cell hybrids generated from CL Brener epimastigotes overexpressing RAD51 – in which HR is increased – was also evaluated, showing that, although not being tetraploid, fused-cell hybrids undoubtedly presented higher DNA content than wild-type cells (Fig. 7). Interestingly, Lewis *et al*. demonstrated that prolonged maintenance of fused-cell hybrids in axenic cultures leads to a gradual and continuous DNA erosion^63^. In fact, since RAD51-overexpressing fused-cell hybrids clones exhibit decreased cellular growth arrest after being irradiated (Fig. 8B), we can suggest that overexpression of RAD51 in *T. cruzi* promotes a more efficient HR, process by which DNA content of fused-cell hybrids can be reduced, promoting genomic stabilization. However, it is expected that other factors also play a role in stabilization of fused-cell hybrids given their decreased growth rate and lower biomass formation (Fig. 8A).

HR is one major source of variability in unicellular organisms, allowing them to acquire beneficial characteristics which can be exploited to promote increased cellular adaptation and survival^64^. In fact, the HR pathway is shown to be activated by the absence of mismatch DNA repair in *Escherichia coli*^47^, and is directly linked to VSG switch in *T. brucei*^55,65^. This suggests that the genetic exchange in naturally-occurring hybrid *T. cruzi* strains is dependent on an efficient mechanism of HR which allows the stabilization of parasexually-originated tetraploid cells^64^; this could explain why it is difficult to detect natural hybrids from naturally-occurring non-hybrid strains. Despite the fact that *T. cruzi* harbors the DNA meiotic recombinase DMC1 – which potentially allows this parasite to produce gametes and perform sexual genetic exchange^66,67^ –, all evidence available so far suggest that *T. cruzi* hybrids are formed by the fusion of individuals which present an increased efficiency to perform HR, thus characterizing parasexual reproduction, a process independent of meiosis^28^. This hybridization can be observed at a generational scale, and it is able to generate tetraploid cells, whose ploidy will decrease through HR, granting the fused-cell hybrids genomic stability and genetic variability.

## Methods

### Cellular strains and culture

Epimastigotes of *T. cruzi* clones CL Brener, Esmeraldo, Sylvio, Dm28c, Y – all strains provided by Dr. Egler Chiari, Universidade Federal de Minas Gerais – and Bug2149 cl10 – a strain kindly provided by Dr. Bianca Zingales, Universidade de São Paulo, Brazil – were maintained in liver infusion tryptose (LIT) medium supplemented with 10% complement-inactivated fetal bovine serum (FBS; Gibco), 200 units/mL penicillin, and 200 µg/L streptomycin sulfate, at 28 °C. Epimastigotes transfected with the integrative vector pROCK_hygroB overexpressing RAD51 were previously obtained^35^, and those harboring the integrative vector pROCK_neo and pROCK_hygroB were transfected according to DaRocha *et al*.^68^. Transfected parasites were cultured in LIT medium supplemented with 200 μg/mL hygromycin or neomycin. Rhesus monkey kidney (LLC-MK2) cells^69^ monolayers were cultured in 2% FBS, 1% penicillin-streptomycin, and 2 mM glutamine supplemented Dulbecco’s Modified Eagle’s Medium (DMEM; Sigma Aldrich), and were infected using axenic *T. cruzi* cultures at stationary phase containing both epimastigotes and metacyclic trypomastigotes. Once the infection was successfully established, trypomastigotes were maintained in LLC-MK2 monolayers and purified by pellet incubation for 2 h at 37 °C, followed by harvest of motile infective cells in the supernatant.

### *T. cruzi* irradiation and cellular growth curve

In order to determine *T. cruzi* response to DSBs generated by ionizing radiation, 5 mL of cellular cultures containing 1.10^7^ parasites/mL were exposed to gamma radiation for 20 min, under a rate of irradiation of 1500 Gy/h using a ^60^Co irradiator, to a total dose of 500 Gy. Cell counting was performed using a Neubauer chamber using erythrosine as vital dye to differentiate live from dead cells. Gamma-irradiated cultures had their cells counted until they reach the stationary growth phase. Experiments were performed in triplicate.

### RNA extraction from *T. cruzi* and real-time quantitative PCR

RNA from *T. cruzi* was purified using the TRIzol Reagent (Invitrogen). Treatment with TURBO DNA-Free Kit (Ambion, Thermo Fisher) was conducted according to manufacturer’s protocol to remove contaminant DNA. PCRs with total RNA were performed with qPCR primers to verify the persistence of any DNA contaminant. A number of 10^8^ parasites were used for RNA extraction at time points 0, 24, and 48 h after exposure to 500 Gy of ionizing radiation – naturally, control cells were not irradiated. The mass of 1 µg of purified RNA was used to cDNA synthesis in a reaction using the High Capacity cDNA Reverse Transcription Kit (Life Technologies), according to manufacturer’s protocol. A reaction with no transcriptase was performed as negative control. Relative quantification of transcripts was performed by the use of specific primers which hybridizes to specific regions of BRCA2 and RAD51 (Table S1). Reactions were conducted in the presence of SYBR Green PCR Master Mix (Applied Biosystems) using glyceraldehyde 3-phosphate dehydrogenase gene (GAPDH) as a control gene. For each reaction were used 2 ng of cDNA, 300 nM of primers, 5 µL of SYBR Green Master Mix 2×, and DNase/RNase free water to 10 µL of reaction using a 384-well plate. All determinations were performed in triplicate. The relative amount of RAD51 transcripts was calculated by the 2^−ΔΔCt^ method.

### Genetic exchange determination in *T. cruzi*

Exponential cultures (~1.10^7^ parasites/mL) of *T. cruzi* were divided in two tissue culture flasks and, each one was labeled with thymidine analogues CldU and IdU 100 µM (Sigma) for 12 h, at 28 °C. Cells were then collected by centrifugation (1500 × *g*, 5 min) and washed 3 times using fresh LIT medium to remove unincorporated thymidine analogues. Parasites were then mixed and incubated for 24 h, at 28 °C, to allow cells undergo genetic exchange. CldU- and/or IdU-labeled cells were washed with 1X PBS (137 mM NaCl, 2.7 mM KCl, 10 mM Na_2_HPO_4_, and 2 mM KH_2_PO_4_, pH 7.4), and fixed in 4% (v/v) paraformaldehyde in 1X PBS, for 10 min, at room temperature. Cells were then deposited on poly-L-lysine coated slides, and permeabilized with 0.1% Triton-X 100 in 1X PBS, for 10 min. Fixed parasites were next washed 3 times with 1X PBS, and treated with 2.5 M HCl, for 20 min, at room temperature, to increase antibody’s accessibility to incorporated thymidine analogues. After that, cells were incubated for 2 h, at room temperature, with rat anti-CldU monoclonal antibody (Accurate), and mouse anti-IdU monoclonal antibody (BD), both diluted 1:300 in blocking solution [4% (w/v) bovine serum albumin diluted in 1X PBS]. Slides were then washed with 1X PBS, and incubated for 2 h, at RT, with secondary antibodies Alexa Fluor 568-conjugated goat anti-mouse (Thermo Scientific), and Alexa Fluor 488-conjugated goat anti-rat (Thermo Scientific), being further washed three times using 1X PBS and VECTASHIELD Mounting Medium with DAPI (Vector Labs) was used as the anti-fade mounting solution, and also to stain nuclear and kinetoplast DNA. Images were captured using Olympus BX51 fluorescence microscope and Olympus XM10 digital camera. Images were analyzed using Olympus Cell^F^ software (version 5.1.2640).

### Selection of *T. cruzi* harboring co-resistance against hygromycin B and neomycin

*T. cruzi* axenic culture was conducted by pooling together, in the same culture flask, a population of CL Brener epimastigotes carrying a vector co-harboring RAD51 and the hygromycin B resistance gene with another population of CL Brener epimastigotes harboring an empty vector carrying the neomycin resistance gene – a flask containing a pool of a population of *T. cruzi* transfected with an empty vector carrying the neomycin resistance gene and another population of *T. cruzi* transfected with an empty vector carrying the hygromycin B resistance gene was used as control. After 21 d of incubation in LIT, this pool of mixed cells was then passaged to LIT supplemented with both hygromycin B and neomycin, and cultured for 60 d in order to select fused-cell hybrids which were resistant to both antibiotics. Rhesus monkey kidney (LLC-MK2)^69^ cells were infected with the same pools of cultures aforementioned described. Aliquots from the supernatant were periodically collected between days 14 and 21, and parasites were then cultured in axenic cultures with LIT supplemented with both hygromycin B and neomycin. Third instar nymphs of *Rhodnius prolixus* were infected with the two previously mentioned pools of populations of CL Brener epimastigotes through artificial feeding. Thirty days after infection, insects were dissected, parasites were isolated, and next cultured in axenic cultures carried out in LIT supplemented with both hygromycin B and neomycin. *T. cruzi* cells showing co-resistance against both hygromycin B and neomycin were plated onto solid blood agar plates supplemented these two antibiotics. After 6 weeks, isolated colonies were recovered from the plates, and inoculated into LIT supplemented with hygromycin B and neomycin.

### *T. cruzi* genomic DNA extraction

*T. cruzi* genomic DNA was extracted through cellular lysis, deproteination and precipitation, as described in Andrade *et al*., 1999^70^. Briefly, a defined number of exponentially-grown *T. cruzi* cells (1 × 10^8^) were washed three times with PBS, and incubated in 200 μL of lysis solution [(0.5% SDS, 100 μM EDTA, and 10 mM Tris-HCl (pH 8.0)] with 20 μg/mL RNase, for 1 h, at 37 °C. Then, 100 μg/mL proteinase K was added to the lysate, which was incubated at 50 °C for 3 h. Deproteination was conducted by the addition of 200 μL saturated phenol followed by gently homogenization, centrifugation, and dispose of the organic phase – the same procedures were repeated for the addition of 200 μL of phenol/chloroform 1:1 (v/v), and 200 μL of chloroform. DNA precipitation was carried out using absolute isopropanol at −80 °C overnight. The isopropanolic suspension of DNA was then centrifuged at 16,000 × *g*, for 10 min, and pelleted DNA was washed twice with ethanol 70% before being dry and resuspended in sterile MilliQ water.

### Determination of survival rates after co-treatment with hygromycin B and neomycin

Survival rates of *T. cruzi* treated with 600 μg/mL hygromycin and/or neomycin were assessed 72 h after antibiotic treatment in LIT medium. The number of live and dead parasites was determined using a Neubauer using erythrosine as vital dye as described previously. All experiments were performed in triplicate.

### Determination of DNA content in *T. cruzi*

The number of 1.10^7^ *T. cruzi* cells at exponential growth phase was collected by centrifugation at 900 × *g*, for 10 min. The supernatant was discarded, and the cellular pellet was washed once in 1X PBS, centrifuged again, being the pellet re-suspended in 70% (v/v) ethanol. After overnight incubation, fixed cells were centrifuged at 900 × *g*, for 10 min, and the supernatant was discarded. The cellular pellet was then washed in PBS, and cells were finally re-suspended in PBS solution containing propidium iodide and RNaseA in final concentrations of 10 µg/mL. Next, samples were incubated for 30 min, at 37 °C, protected from light, and their DNA content was measured by FACSCan™ flow cytometer (BD), and analized using FlowJo software.

## Electronic supplementary material

Supplementary Data
