# Supplementary material for: The recombinase Rad51 plays a key role in events of genetic exchange in Trypanosoma cruzi
Source: Sci Rep. 2018 Sep 6;8:13335. doi: 10.1038/s41598-018-31541-z (PMC6127316; doi:10.1038/s41598-018-31541-z)
Supplement: Supplementary file 1 — Supplementary Data [file 41598_2018_31541_MOESM1_ESM.pdf]

The recombinase Rad51 plays a key role in events of genetic exchange in *Trypanosoma cruzi*

Ceres Luciana Alves[1*], Bruno Marçal Repolês[1*], Marcelo Santos da Silva[2*], Isabela Cecília Mendes[1], Paula Andrea Marin[2], Pedro Henrique Nascimento Aguiar[1], Selma da Silva Santos[1], Glória Regina Franco[1], Andréa Mara Macedo[1], Sérgio Danilo Junho Pena[1], Luciana de Oliveira Andrade[3], Alessandra Aparecida Guarneri[4], Erich Birelli Tahara[1], Maria Carolina Elias[2], Carlos Renato Machado[1*]

1 Departamento de Bioquímica e Imunologia, Universidade Federal de Minas Gerais, Belo Horizonte, MG, Brazil

2 Laboratório Especial de Ciclo Celular, Centro de Toxinas, Resposta Imune e Sinalização Celular, Instituto Butantan, São Paulo, Brazil

3 Departamento de Morfologia, Universidade Federal de Minas Gerais, Belo Horizonte, MG, Brazil

4 Centro de Pesquisas René Rachou, FIOCRUZ, Belo Horizonte, MG, Brazil

* These authors contributed equally to the work

Corresponding Author

Carlos Renato Machado

Departamento de Bioqumica e Imunologia, ICB, UFMG Av. Antonio Carlos, 6627, Caixa postal 486, Belo Horizonte, MG Brazil.

E-mail: crmachad@icb.ufmg.br

SUPPORTING INFORMATION LEGENDS

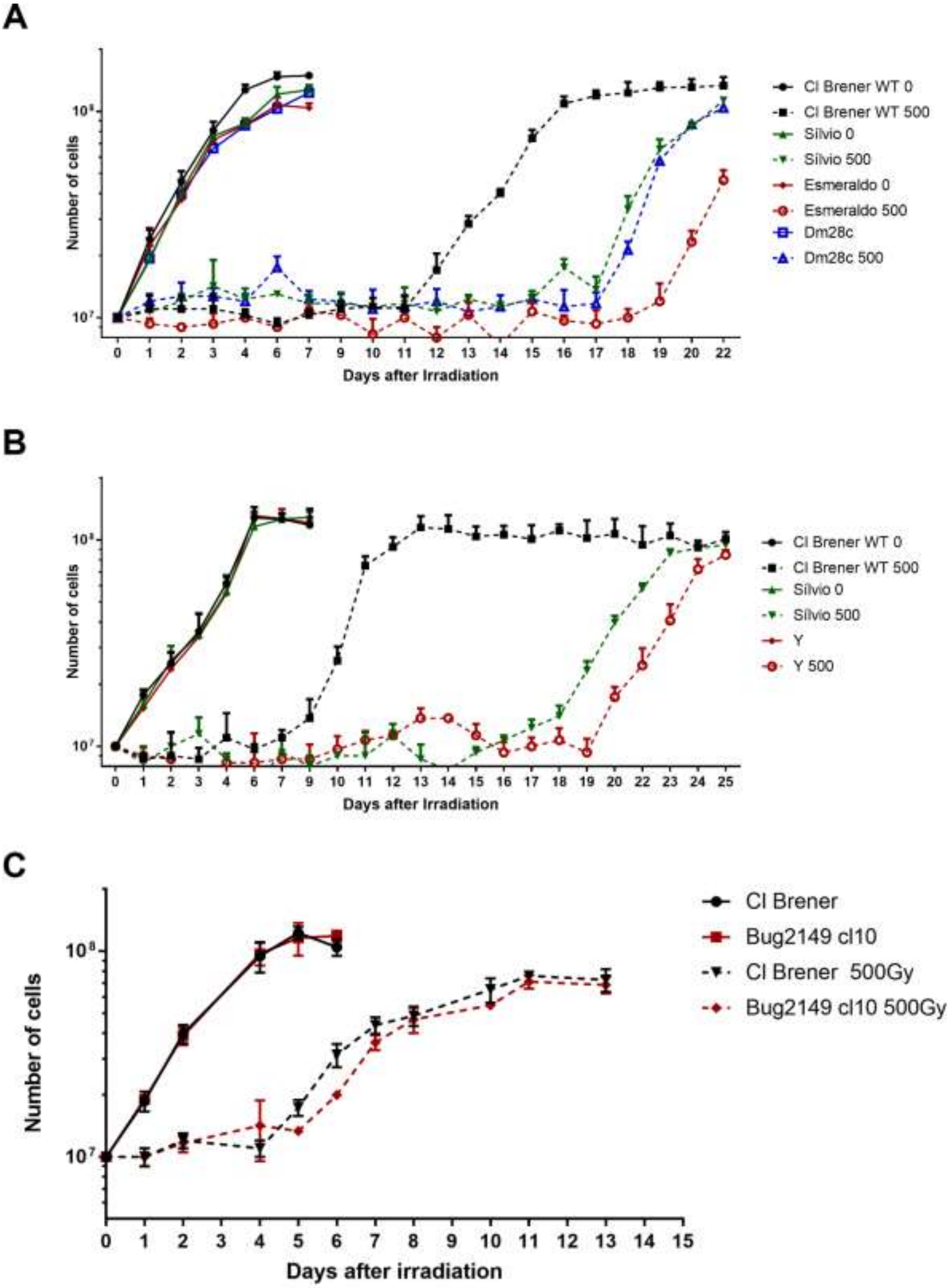

S1 Fig. Determination of cellular growth arrest in different strains of *T. cruzi*. *T. cruzi* epimastigotes were exposed to 500 Gy of ionizing radiation. **A:** Dm28c strain, a naturally-occurring non-hybrid cell (TcI), presents increased cellular growth arrest after irradiation. **B:** Y strain, a naturally-occurring non-hybrid cell (TcII), also presents increased cellular growth arrest after irradiation. **C:** TcBug strain, a naturally-occurring hybrid cell (TcV), resumes its growth simultaneously with CL Brener WT strain.

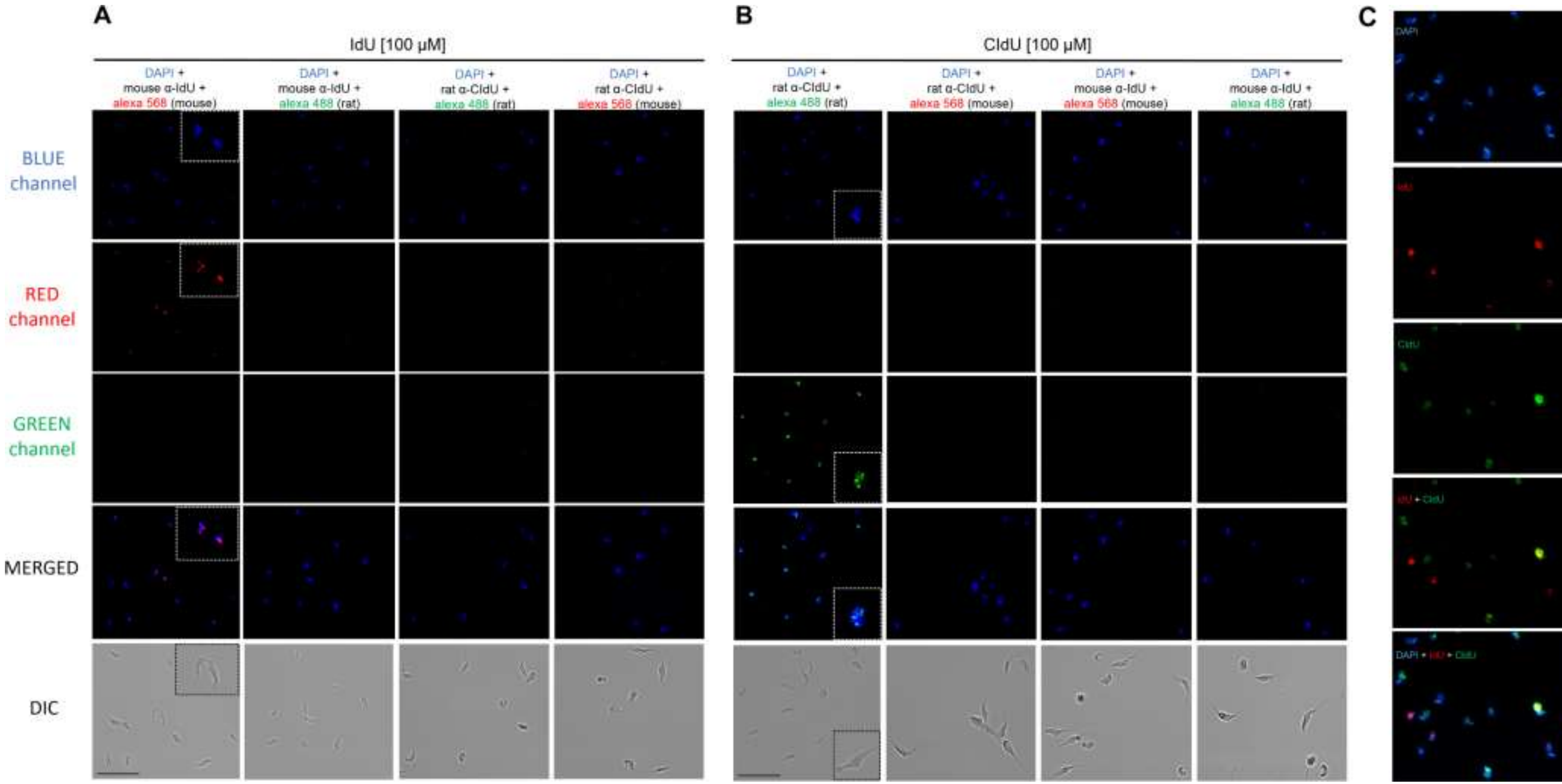
A
IdU [100 µM]
DAPI + mouse α-IdU + alexa 568 (mouse)
DAPI + mouse α-IdU + alexa 488 (rat)
DAPI + rat α-CldU + alexa 488 (rat)
DAPI + rat α-CldU + alexa 568 (mouse)
B
CldU [100 µM]
DAPI + rat α-CldU + alexa 488 (rat)
DAPI + rat α-CldU + alexa 568 (mouse)
DAPI + mouse α-IdU + alexa 568 (mouse)
DAPI + mouse α-IdU + alexa 488 (rat)
C
BLUE channel
RED channel
GREEN channel
MERGED
DIC
DAPI
IdU
CldU
IdU + CldU
DAPI + IdU + CldU

33

S2 Fig. The recognition thymidine analogues is specific. Epimastigotes of *T. cruzi* clone CL Brener were incubated in the presence of thymidine analogues for 12 h. **A:** IdU-incorporated epimastigotes were added onto slides, and processed for detection using α-CldU and the corresponding secondary antibody (Alexa Fluor 488). Small squares show representative images of each channel observed in the large square (merged), which is the superimposition of DAPI + green + red channels. We observed complete absence of cross-reactions. **B**. CldU-incorporated epimastigotes were added onto slides and processed in the same manner as previously described, but using α-IdU and the corresponding secondary antibody (Alexa Fluor 555). As previously indicated, small squares show representative images of each channel observed in the large square (merged), which is the superposition of DAPI + green + red channels. We observed again complete absence of cross-reactions. Images were analysed using Olimpus BX51 fluorescence microscope, and captured randomly. N: nucleus; K: kinetoplast. Bar represents 200 µm. **C**. Secondary antibodies specifities test: as demonstrate on the figure, there's no cross detection among the antibodies used on the experiment. The secondary antibody for one thymidine analog do not present any detection with the secondary antibody for the other analog. From up to down: DAPI staining; IdU-incorporated detection using α-IdU, and processed for detection using the respective secondary antibody (Alexa Fluor 555); ClU-incorporated detection using α-CldU , and processed for detection using the respective secondary antibody (Alexa Fluor 498); Double detection of both analogs by using the secondary antibodies for CldU and IdU; DAPI+ αIdU Alexa Fluor 555 + αCldU Alexa Fluor 488 merge.

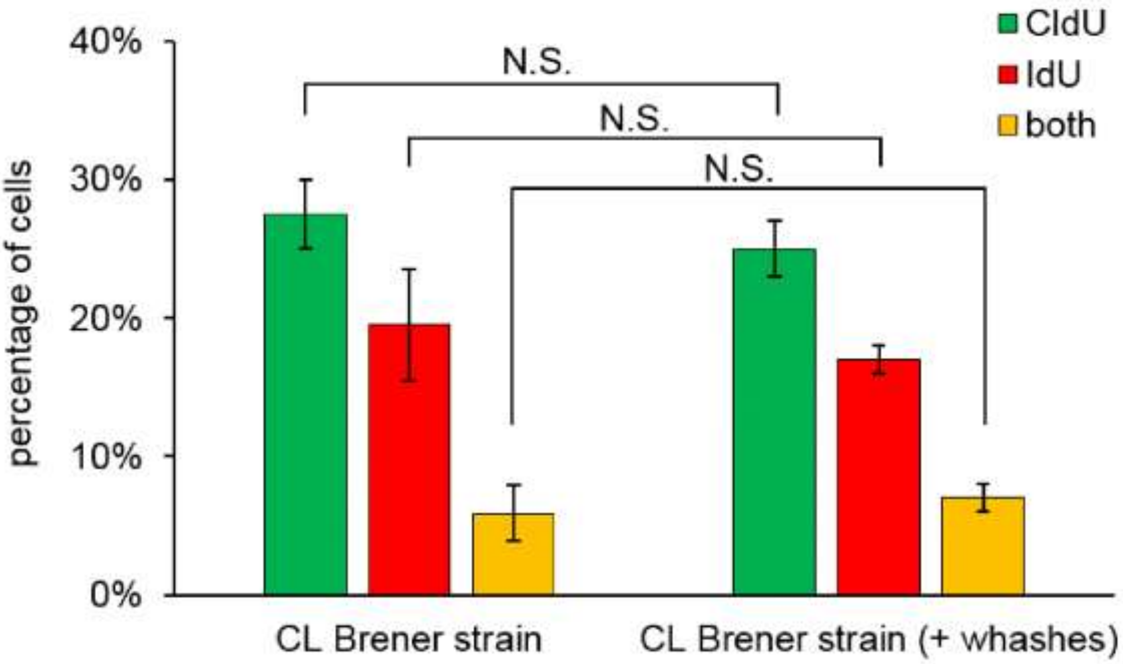
CldU
IdU
both
40%
30%
20%
10%
0%
percentage of cells
N.S.
N.S.
N.S.
CL Brener strain
CL Brener strain (+ whashes)

S3 Fig. Washes increasing the buffer volume do not alter significantly the percentage of labeled cells. Percentage of epimastigotes of *T. cruzi* CL Brener labeled with CldU (green), IdU (red), or with both analogues (yellow) did no show significant alterations after increasing the wash buffer volume from 1 mL each wash to 15 mL each wash (+ washes). n = 335 cells for CL Brener and n = 341 cells of CL Brener (+ washes). Experiments were carried out in triplicate, and error bars indicate standard deviation (SD). Statistical analysis was performed using Student's *t*-test.
